# Supplementary material for: Language models can learn complex molecular distributions
Source: Nat Commun. 2022 Jun 7;13:3293. doi: 10.1038/s41467-022-30839-x (PMC9174447; doi:10.1038/s41467-022-30839-x)
Supplement: Supplementary file 1 — Supplementary Info [file 41467_2022_30839_MOESM1_ESM.pdf]

Supplementary Information: Language Models can learn Complex Molecular Distributions

D. Flam-Shepherd et al.

Supplementary Discussion We experiment with additional graph generative model baselines on all tasks. These include HierVAE [1], GCPN [2], GRAPH AF [3], GENRIC [4], CNF [5], Molecular RNN (MRNN) [6], GRAPHVAE [7], NAT-GRAPHVAE [8], MOLGAN [9], GRAPH NVP [10], DGMG [11], MOLMP [12], GRAPHINVENT [13]. From these models, most of the single shot generative models do not scale– from MOLGAN, GRAPHVAE, NAT-GRAPHVAE, GRAPH NVP. None of these models including GCPN were able to acheive better than 1% valid, unique and novel– meaning they are unable to generate molecules from the training distribution. Furthermore, all of the autoregressive graph generative models (DGMG, MOLMP, GRAPH INVENT) were unable to handle the larger molecules– even in the LogP and Multi-distribution tasks. Training on these larger datasets exacerbate the stability issues [11] these models suffer from– making them unable to stably train to completion. The baselines that were able to train could only handle the LogP and multi-distribution tasks, these include: two discrete normalizing flow models CNF [5] and GRAPH AF [3], GENRIC which employs a Markov chain, MRNN or Molecular RNN which uses RNNs to generate atoms and bonds and HIERVAE which extends JTVAE to larger common motifs or substructures. All baselines have high scoring standard metrics (Table II) but their wasserstein distance metrics are much further from the Train Oracle than the RNNs (Table I). HIERVAE and MRNN stand out and are higher scoring than GENRIC, CNF and GRAPH AF– HIERVAE even beats the SF-RNN on SA and NP but not the SM-RNN. Indeed, from the distribution plot in Figure 1**a** for the LogP task we can see that MRNN and HIERVAE are closer to the training distribution than the additional baselines but nearly as close as the RNNs. For the multi-distribution task, the closest are MRNN and CNF, shown in the distribution plot in Supplementary Fig. 1**c**– where MRNN learns all of the modes (but poorly) while CNF entirely misses the CEP mode. In contrast the RNNs, perfectly learn all four modes (Supplementary Fig. 1**b**).

Task	Samples	LogP	SA	QED	MW	BCT	NP
LogP	TRAIN	0.020	0.0096	0.0029	1.620	7.828	0.013
	SM-RNN	0.095	0.0312	0.0068	3.314	21.12	0.054
	SF-RNN	0.177	0.2903	0.0095	6.260	25.00	0.209
	HIERVAE	0.661	0.0464	0.0710	51.73	141.9	0.079
	MRNN	0.769	1.2321	0.0710	58.27	142.9	0.898
	GRAPHAF	3.534	1.8820	0.2413	164.7	664.4	1.206
	CNF	2.773	3.4727	0.1879	37.87	174.7	1.456
	GENRIC	2.764	1.3626	0.1092	81.41	308.0	1.286
Multi	TRAIN	0.048	0.0158	0.0020	2.177	14.15	0.010
	SM-RNN	0.081	0.0246	0.0059	5.483	21.19	0.012
	SF-RNN	0.286	0.1791	0.0227	11.35	68.81	0.079
	HIERVAE	2.356	0.2151	0.1024	157.7	687.0	0.175
	MRNN	1.519	0.6644	0.0593	97.92	400.1	0.598
	GRAPHAF	3.140	1.9122	0.1174	106.1	971.7	0.723
	CNF	2.378	2.0793	0.0991	61.87	436.7	1.070
	GENRIC	1.623	2.0029	0.0827	105.7	445.3	0.787

Supplementary Table I. **Wasserstein distance metrics** for LogP, SA, QED, MW, BT and NP between molecules from the training data and generated by the additional baselines and RNNs for all three tasks. Values closer to TRAIN are better.

Task	Metric	SM-RNN	SF-RNN	HIERVAE	MRNN	GRAPHAF	CNF	GENRIC
LogP	validity	0.941	1.000	1.000	1.000	1.000	1.000	1.000
	unique	0.987	1.000	1.000	0.999	0.906	1.000	0.886
	novelty	0.721	0.871	1.000	0.994	1.000	1.000	0.993
Multi	valid	0.969	1.000	1.000	0.999	1.000	1.000	0.997
	unique	0.996	0.989	0.938	0.999	0.985	1.000	0.912
	novelty	0.937	0.950	1.000	1.000	1.000	1.000	0.998

Supplementary Table II. **Standard Metrics.** From molecules generated by all models (Closer to 1.0 is better).

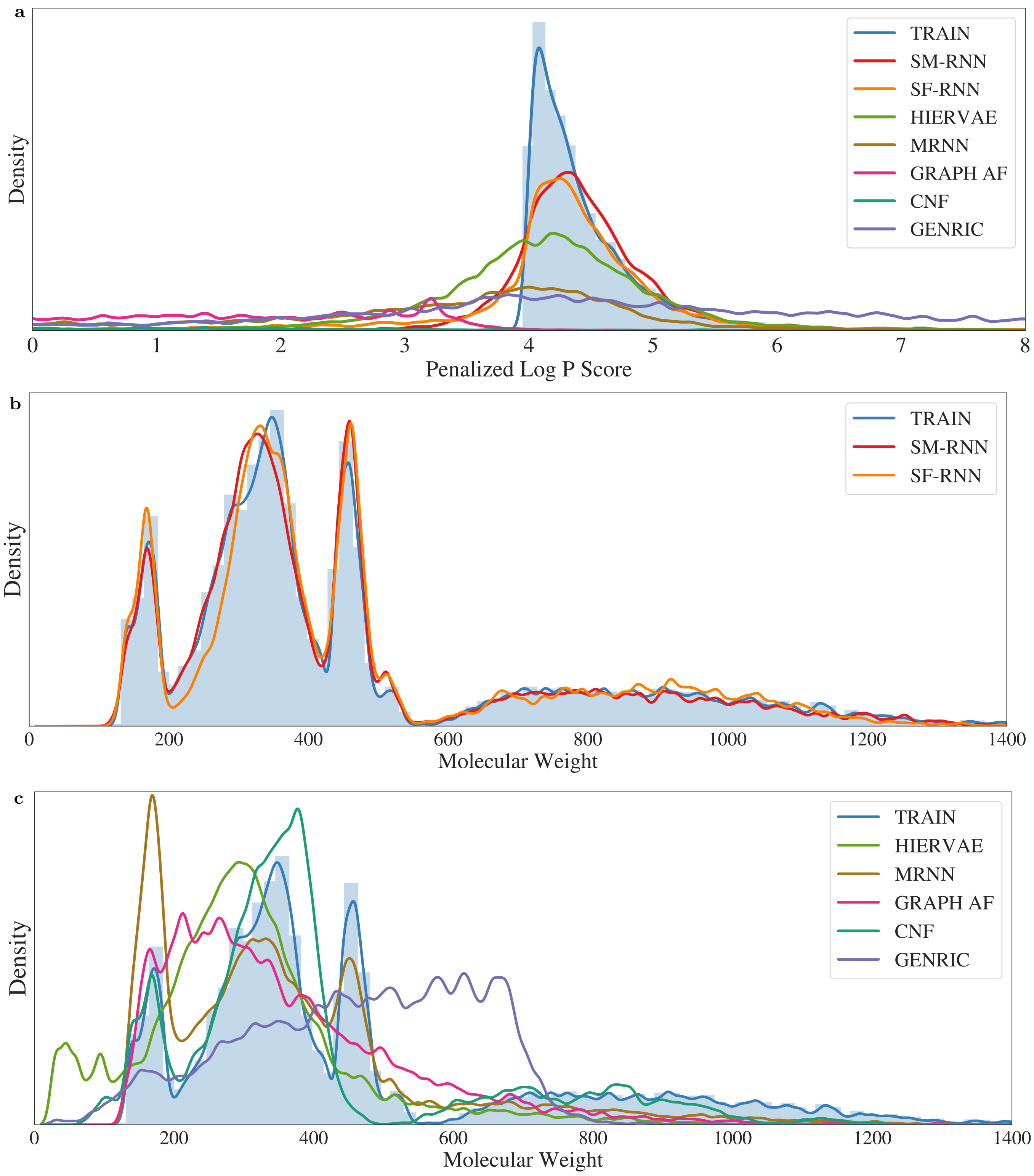

Supplementary Fig. 1. **Additional Baselines a** For the LogP task, the histogram and KDE of penalized logP of training molecules along with KDEs of molecular weight of molecules generated from additional baselines model that could generate samples. **b** The histogram and KDE of molecular weight of training molecules along with KDEs of molecular weight of molecules generated from the training data and RNNs. **c** The histogram and KDE of molecular weight of training molecules along with KDEs of molecular weight of molecules generated from the training data and from additional baselines.

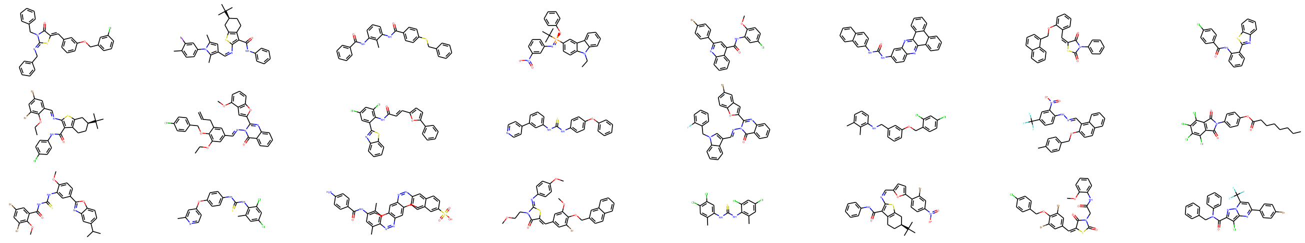

a Molecules with 4 ≤ penalized LogP ≤ 5.

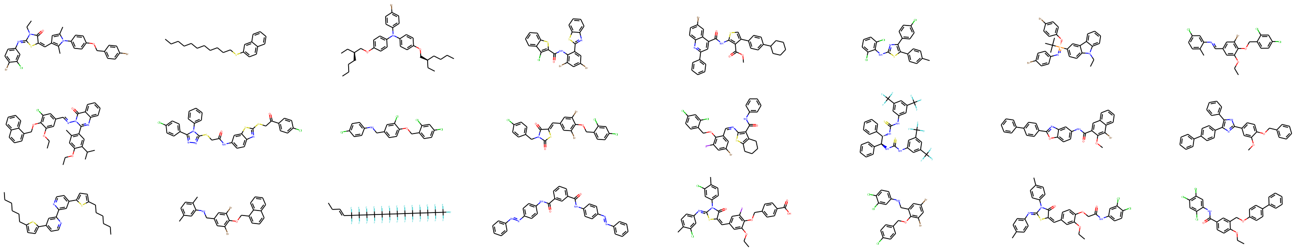

b Molecules with 5 ≤ penalized LogP ≤ 6.

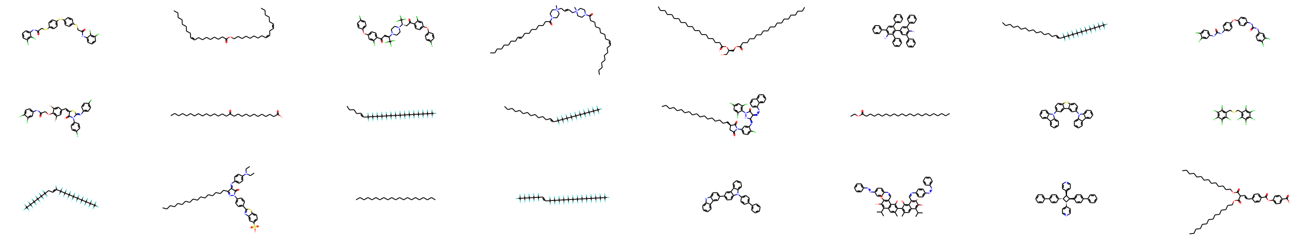

c Molecules with 6 ≤ penalized LogP ≤ 8.

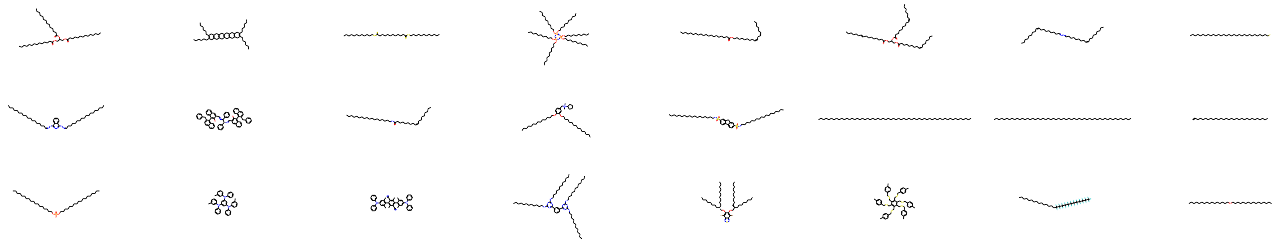

d Molecules with penalized LogP ≥ 8.

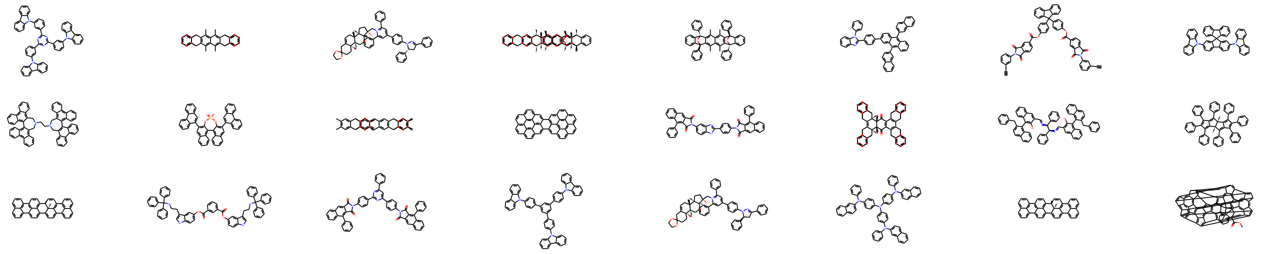

e Molecules with more than 10 rings.

Supplementary Fig. 2. **Penalized LogP Task a-e** Training molecules with different properties.

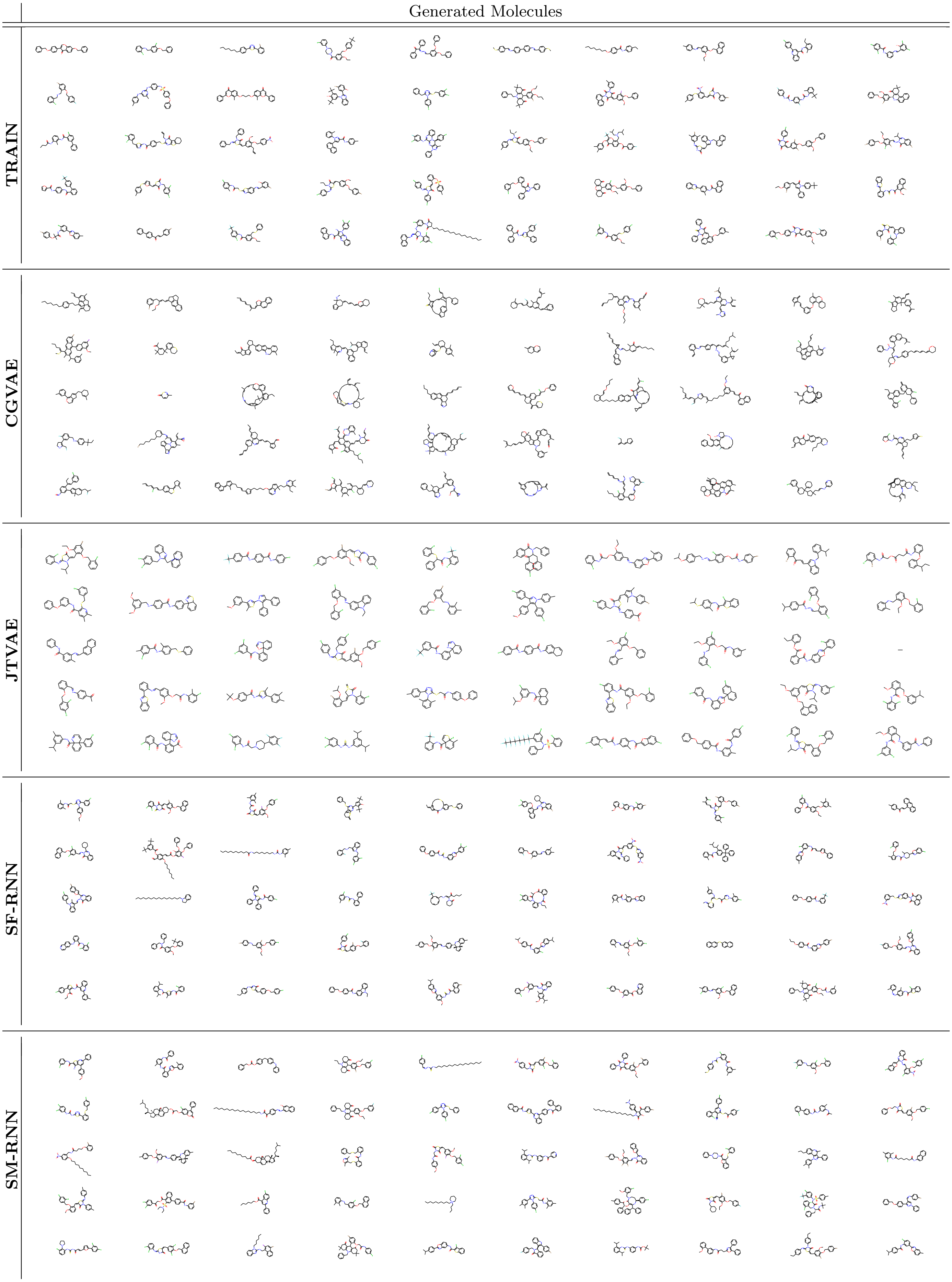

Supplementary Table III. **Penalized LogP Task** Molecules generated from each model.

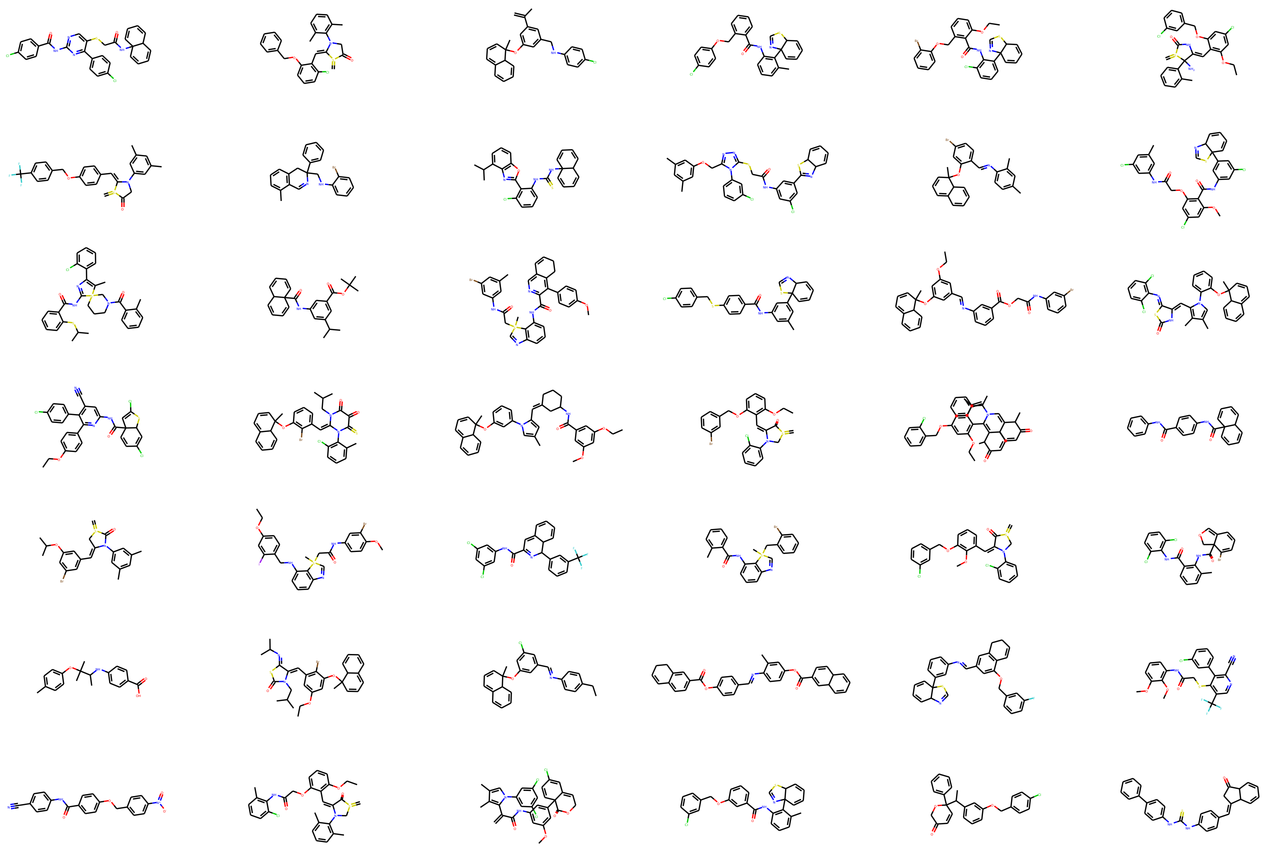

a Molecules from JTVAE.

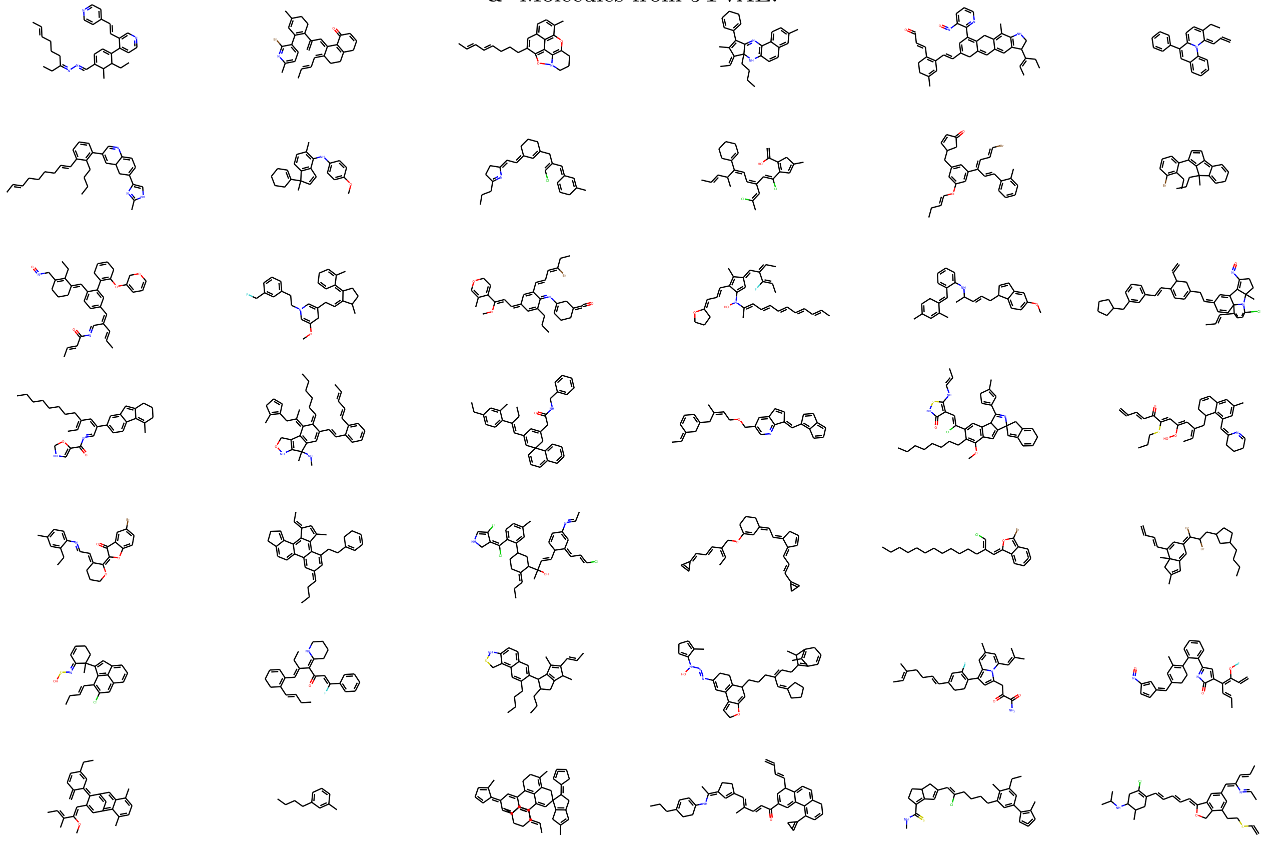

b Molecules from CGVAE.

Supplementary Fig. 3. **Penalized LogP Task a-b** Generated molecules with penalized LogP < 4 from the graph generative models.

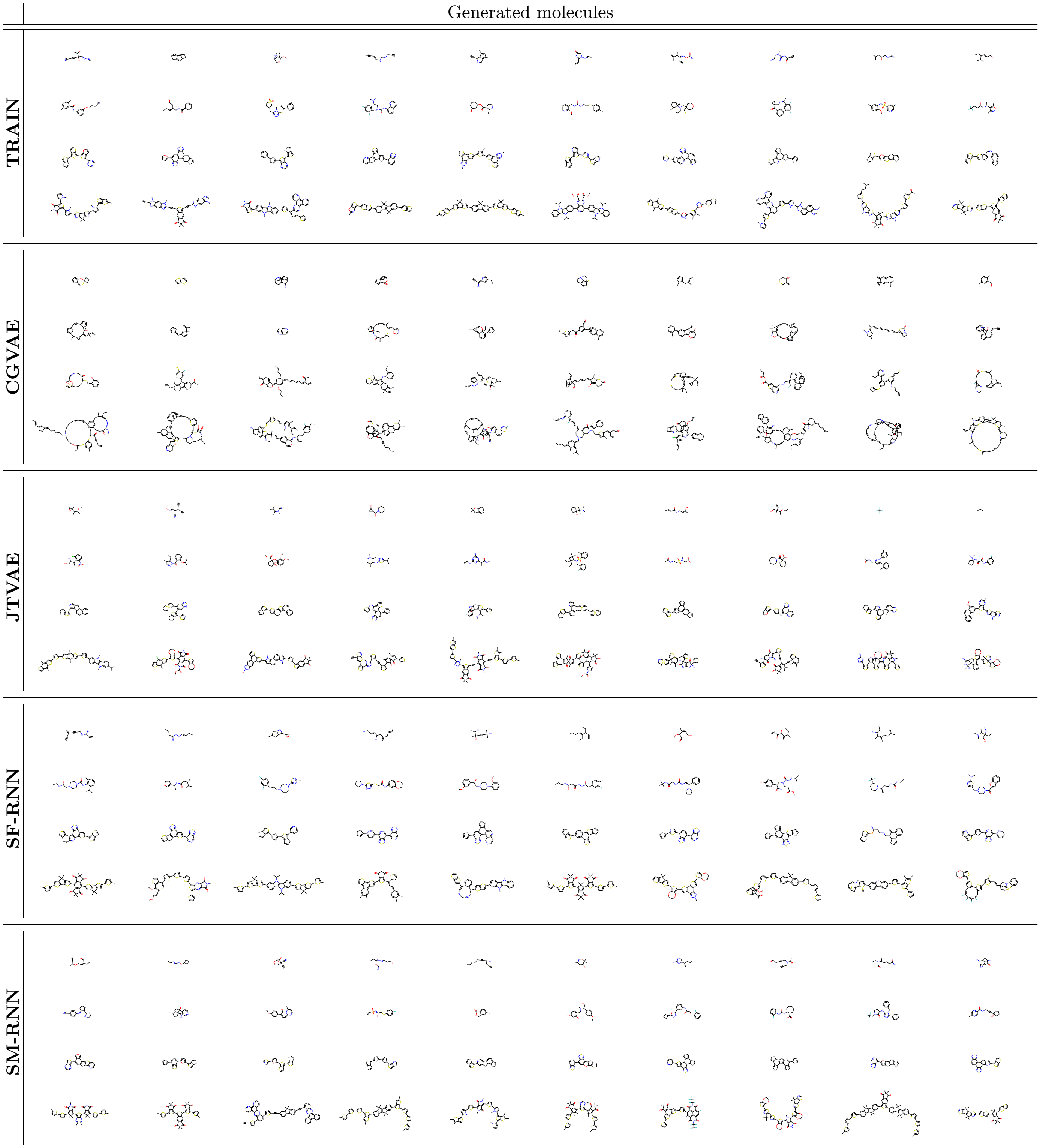

Supplementary Table IV. **Multi-distribution Task** Model generated molecules. Each sub-row is from a specific molecular mode.

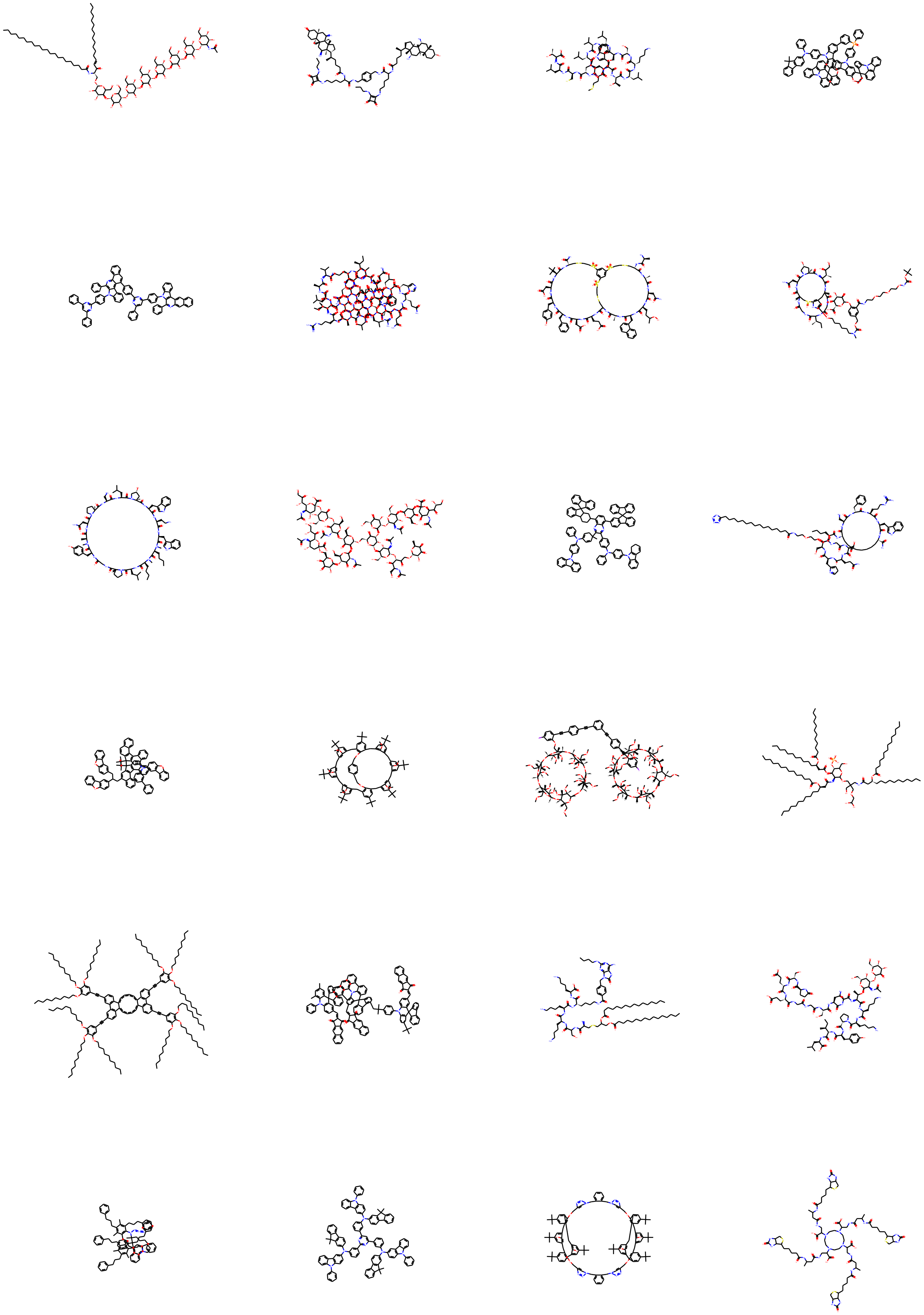

Supplementary Fig. 4. **Large Scale Task** Training molecules.

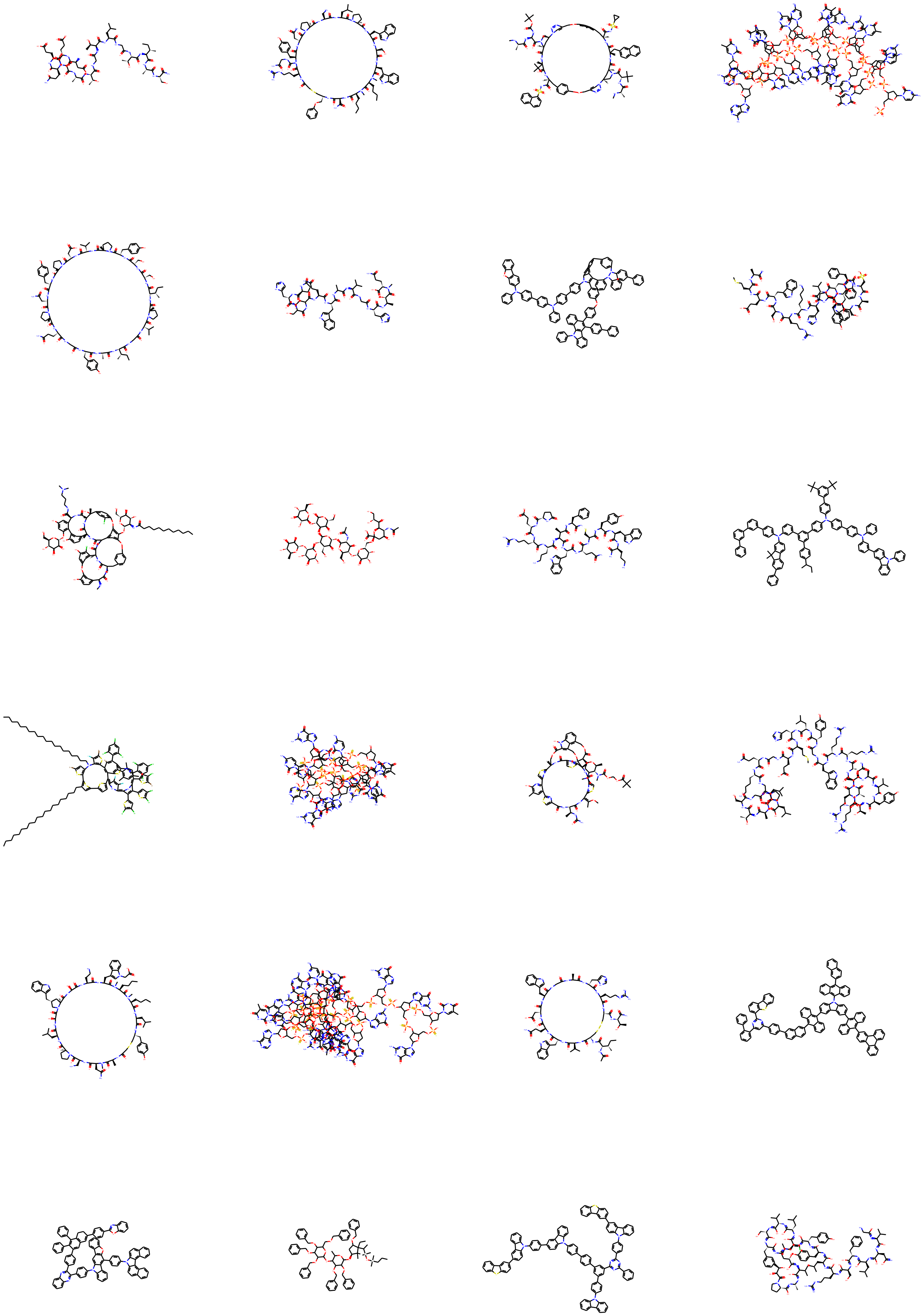

Supplementary Fig. 5. **Large Scale Task** Generated molecules from the SMILES RNN.

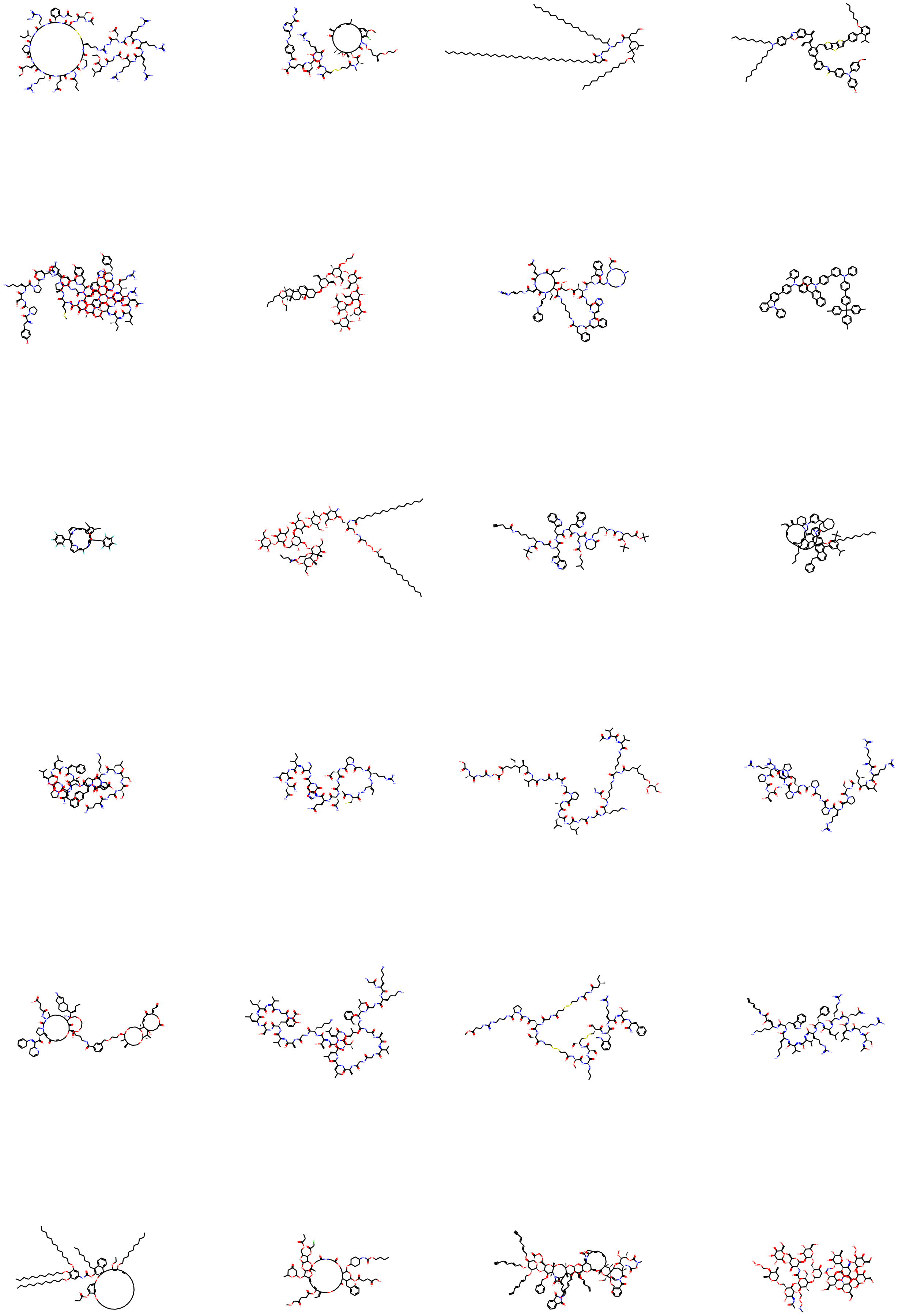

Supplementary Fig. 6. **Large Scale Task** Generated molecules from the SELFIES RNN.

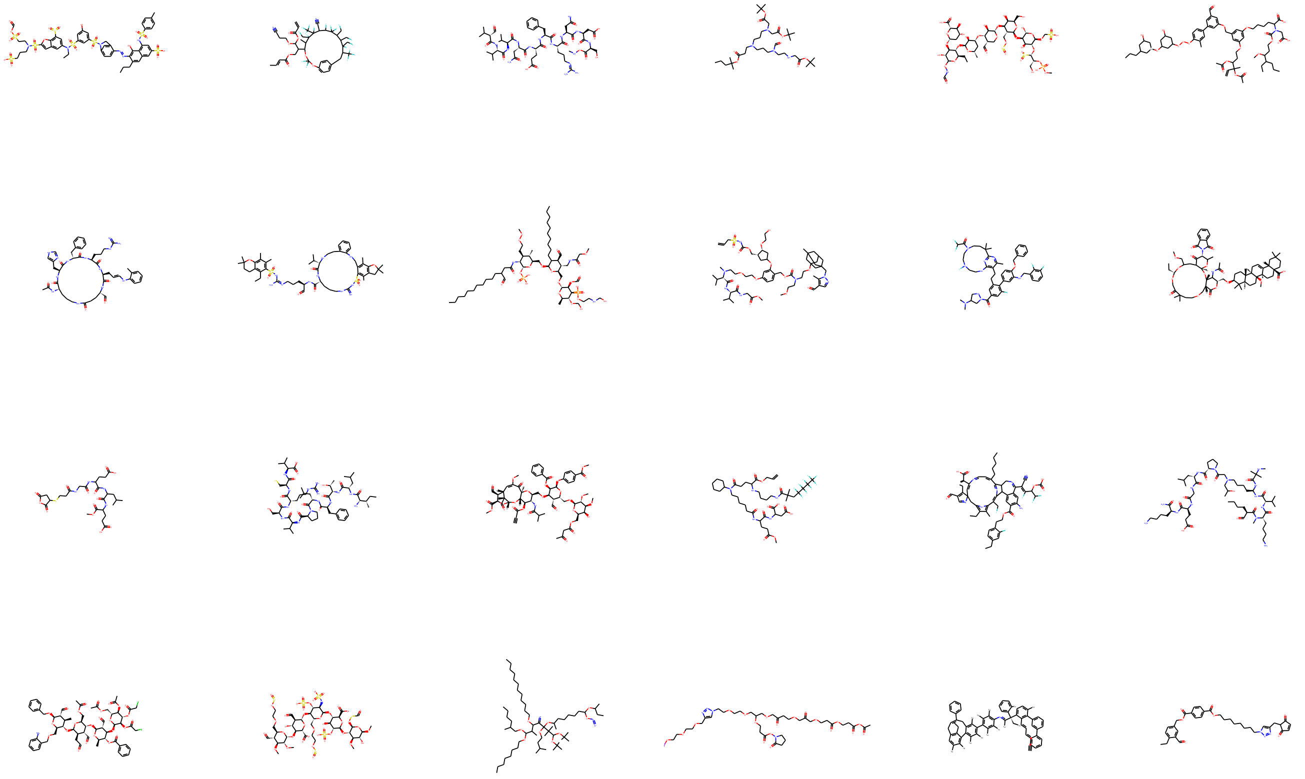

a Molecules from the SELFIES RNN.

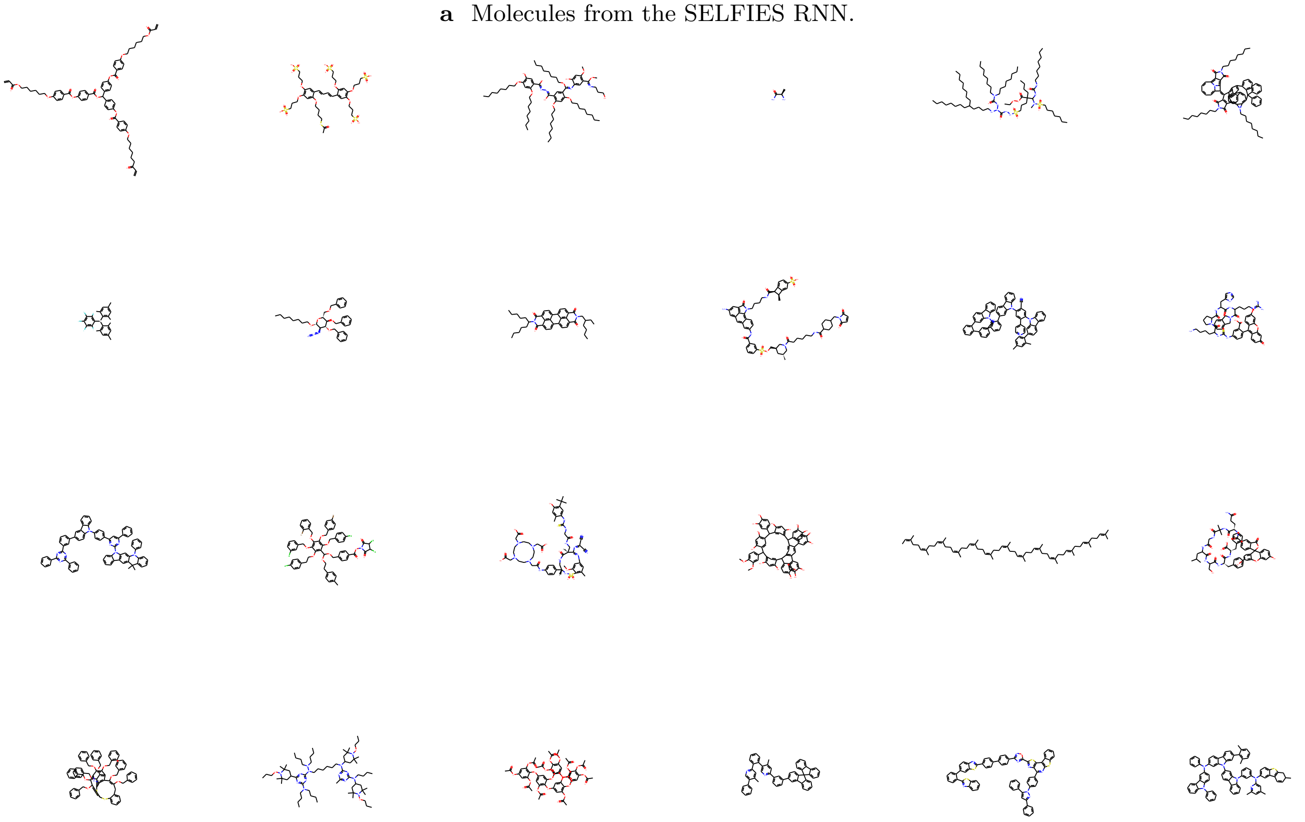

b Molecules from the SMILES RNN.

Supplementary Fig. 7. **Large Scale Task a-b** Generated molecules with less than 100 heavy atoms from the RNN models.

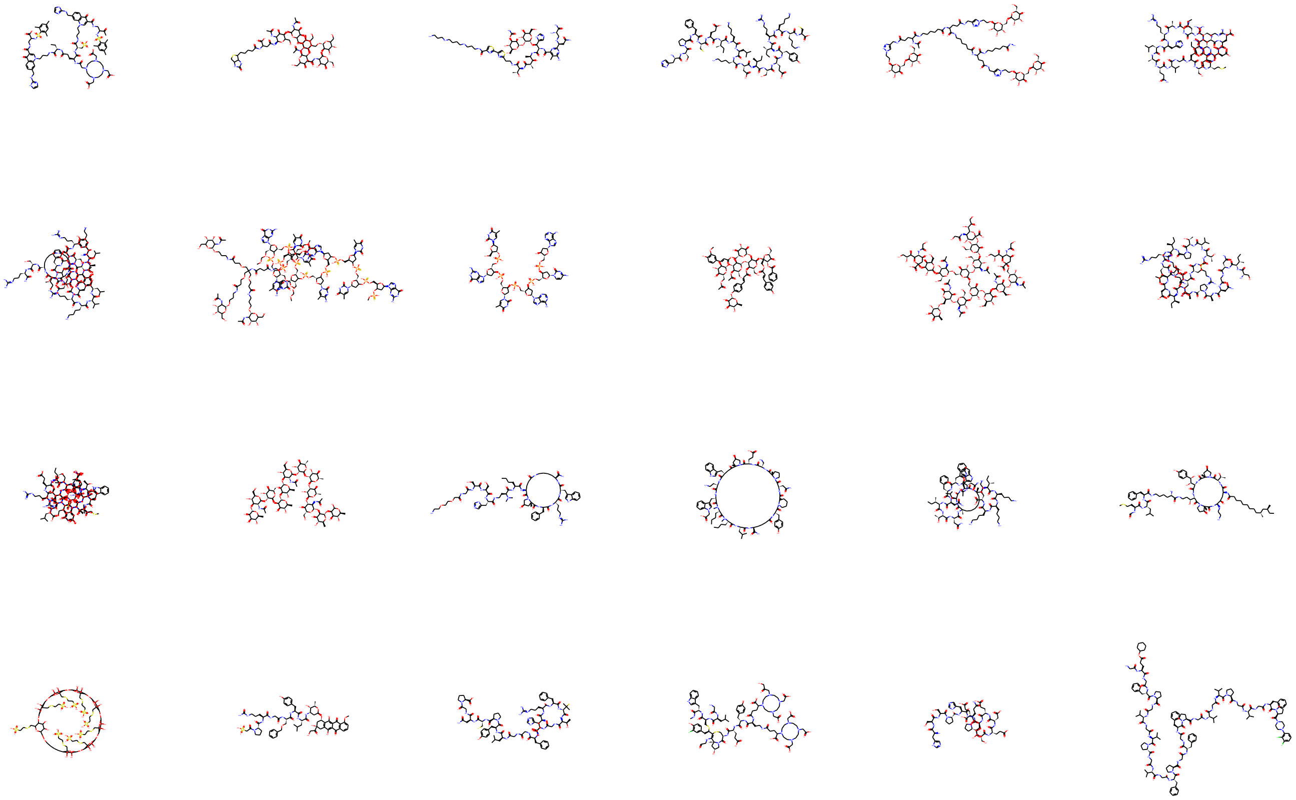

a Molecules from the mode with lower LogP values.

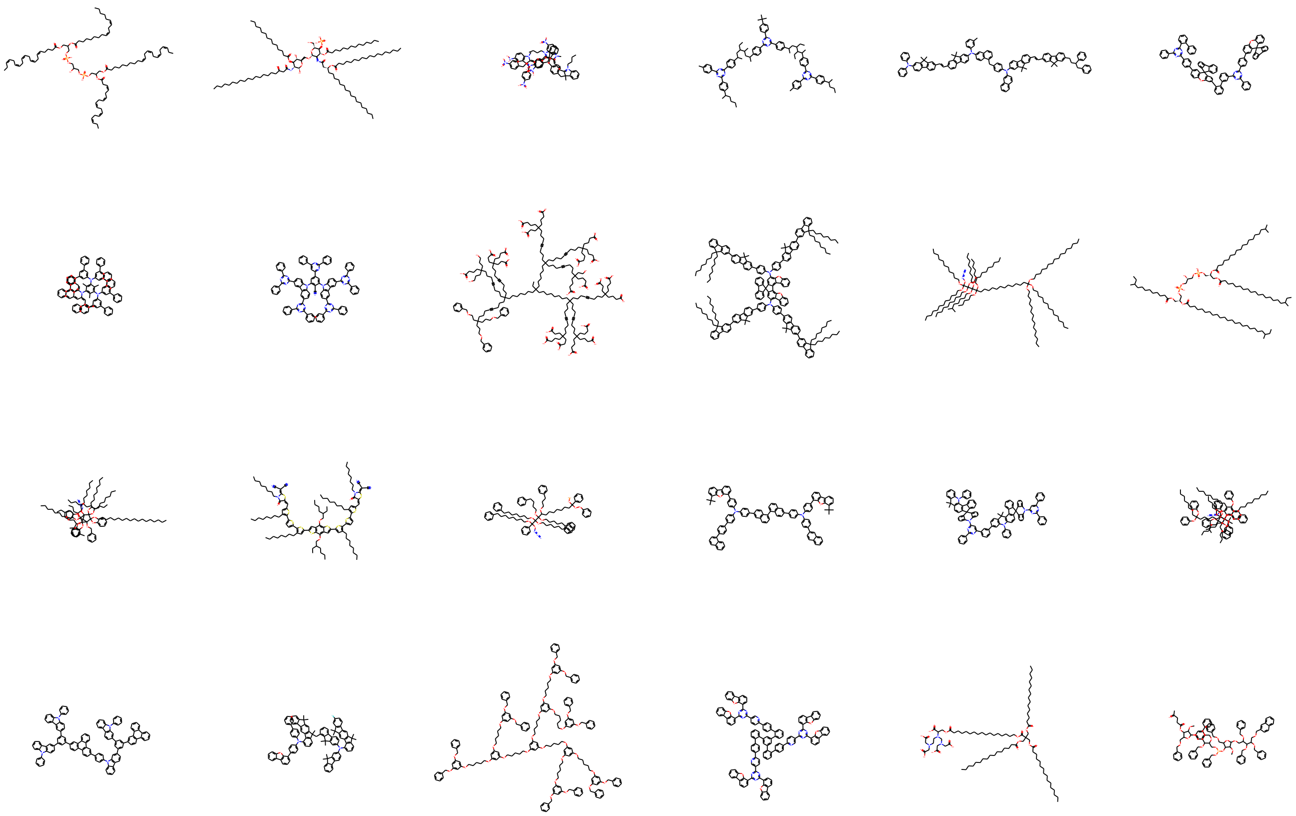

b Molecules from the mode with higher LogP values.

Supplementary Fig. 8. **Large Scale Task a-b** Training molecules from each LogP mode.

SUPPLEMENTARY REFERENCES

[1] Wengong Jin, Regina Barzilay, and Tommi Jaakkola, "Hierarchical generation of molecular graphs using structural motifs," in International Conference on Machine Learning (PMLR, 2020) pp. 4839–4848.

[2] Jiaxuan You, Bowen Liu, Zhitao Ying, Vijay Pande, and Jure Leskovec, "Graph convolutional policy network for goal-directed molecular graph generation," "Graph convolutional policy network for goal-directed molecular graph generation," Advances in Neural Information Processing Systems, , 6410–6421 (2018).

[3] Chence Shi, Minkai Xu, Zhaocheng Zhu, Weinan Zhang, Ming Zhang, and Jian Tang, "Graphaf: a flow-based autoregressive model for molecular graph generation," "Graphaf: a flow-based autoregressive model for molecular graph generation," arXiv preprint arXiv:2001.09382 (2020).

[4] Ari Seff, Wenda Zhou, Farhan Damani, Abigail Doyle, and Ryan P Adams, "Discrete object generation with reversible inductive construction," in Advances in Neural Information Processing Systems.

[5] Phillip Lippe and Efstratios Gavves, "Categorical normalizing flows via continuous transformations," "Categorical normalizing flows via continuous transformations," arXiv preprint arXiv:2006.09790 (2020).

[6] Mariya Popova, Mykhailo Shvets, Junier Oliva, and Olexandr Isayev, "Molecularrnn: Generating realistic molecular graphs with optimized properties," "Molecularrnn: Generating realistic molecular graphs with optimized properties," arXiv preprint arXiv:1905.13372 (2019).

[7] Martin Simonovsky and Nikos Komodakis, "Graphvae: Towards generation of small graphs using variational autoencoders," in International Conference on Artificial Neural Networks (Springer, 2018) pp. 412–422.

[8] Youngchun Kwon, Jiho Yoo, Youn-Suk Choi, Won-Joon Son, Dongseon Lee, and Seokho Kang, "Efficient learning of non-autoregressive graph variational autoencoders for molecular graph generation," "Efficient learning of non-autoregressive graph variational autoencoders for molecular graph generation," Journal of cheminformatics (2019).

[9] Nicola De Cao and Thomas Kipf, "Molgan: An implicit generative model for small molecular graphs," "Molgan: An implicit generative model for small molecular graphs," arXiv preprint arXiv:1805.11973 (2018).

[10] Kaushalya Madhawa, Katushiko Ishiguro, Kosuke Nakago, and Motoki Abe, "Graphnvp: An invertible flow model for generating molecular graphs," "Graphnvp: An invertible flow model for generating molecular graphs," arXiv preprint arXiv:1905.11600 (2019).

[11] Yujia Li, Oriol Vinyals, Chris Dyer, Razvan Pascanu, and Peter Battaglia, "Learning deep generative models of graphs," "Learning deep generative models of graphs," arXiv preprint arXiv:1803.03324 (2018).

[12] Yibo Li, Liangren Zhang, and Zhenming Liu, "Multi-objective de novo drug design with conditional graph generative model," "Multi-objective de novo drug design with conditional graph generative model," Journal of cheminformatics **10**, 1–24 (2018).

[13] Rocío Mercado, Tobias Rastemo, Edvard Lindelöf, Günter Klambauer, Ola Engkvist, Hongming Chen, and Esben Jannik Bjerrum, "Graph networks for molecular design," "Graph networks for molecular design," Machine Learning: Science and Technology **2**, 025023 (2021).
